# Epitope Analysis of the Collagen Type V-Specific T Cell Response in Lung Transplantation Reveals an HLA-DRB1*15 Bias in Both Recipient and Donor

**DOI:** 10.1371/journal.pone.0079601

**Published:** 2013-11-12

**Authors:** Melissa R. Keller, Lynn D. Haynes, Ewa Jankowska-Gan, Jeremy A. Sullivan, Vrushali V. Agashe, Scott R. Burlingham, William J. Burlingham

**Affiliations:** 1 Department of Surgery, University of Wisconsin, Madison, Wisconsin, United States of America; 2 Cellular and Molecular Pathology Graduate Program, University of Wisconsin, Madison, Wisconsin, United States of America; 3 Comparative Biomedical Sciences Graduate Program, University of Wisconsin, Madison, Wisconsin, United States of America; 4 Department of Chemistry, University of Wisconsin, Madison, Wisconsin, United States of America; Xavier Bichat Medical School, INSERM-CNRS - Université Paris Diderot, France

## Abstract

**Background:**

IL-17-dependent cellular immune responses to the α1 chain of collagen type V are associated with development of bronchiolitis obliterans syndrome after lung transplantation, and with idiopathic pulmonary fibrosis and coronary artery disease, primary indications for lung or heart transplantation, respectively.

**Methodology/Principal Findings:**

We found that 30% of the patients awaiting lung transplantation exhibited a strong cell-mediated immune response to col(V). Of these, 53% expressed HLA-DR15, compared to a 28% HLA-DR15 frequency in col(V) low-responders (p=0.02). After transplantation, patients with HLA-DR1 and -DR17, not -DR15, developed anti-col(V) responses most frequently (p=0.04 and 0.01 vs. controls, respectively). However, recipients of a lung from an HLA-DR15^+^ donor were at significantly elevated risk of developing anti-col(V) responses (p=0.02) and BOS (p=0.03). To determine the molecular basis of this unusual pattern of DR allele bias, a peptide library comprising the collagenous region of the α1(V) protein was screened for binding to HLA-DR0101, -DR1501, -DR0301 (DR17) or to HLA-DQ2 (DQA1*0501: DQB1*0201; in linkage disequilibrium with -DR17) and -DQ6 (DQA1*0102: DQB1*0602; linked to -DR15). Eight 15-mer peptides, six DR-binding and two DQ-binding, were identified. HLA-DR15 binding to two peptides yielded the highest binding scores: 650 (where 100 = positive control) for p799 (GIRGLKGTKGEKGED), and 193 for p1439 (LRGIPGPVGEQGLPG). These peptides, which also bound weakly to HLA-DR1, elicited responses in both HLA-DR1^+^ and -DR15^+^ col(V) reactive hosts, whereas binding and immunoreactivity of p1049 (KDGPPGLRGFPGDRG) was DR15-specific. Remarkably, a col(V)-reactive HLA-DR1^+^DR15^neg^ lung transplant patient, whose donor was HLA-DR15^+^, responded not only to p799 and p1439, but also to p1049.

**Conclusions/Significance:**

HLA-DR15 and IPF disease were independently associated with pre-transplant col(V) autoimmunity. The increased risk of de novo immunity to col(V) and BOS, associated with receiving a lung transplant from an HLA-DR15^+^ donor, may result from presentation by donor-derived HLA- DR15, of novel self-peptides to recipient T cells.

## Introduction

Recent studies have suggested a common autoimmune component to fibro-obliterative diseases of lung [1-3], and heart [4,5], and to chronic rejection of lung [6,7] and heart transplants [8,9]. Our group and others have shown that these fibro-obliterative pathologies share the feature of T helper type 17 (Th17)-mediated immunity to collagen type V [col(V)], specific for the alpha-1 chain of col(V) (α1(V)) chain [10-12], and can lead to both acute and chronic tissue injury. End-stage lung disease patients with col(V) immunoreactivity at the time of lung transplantation were at significantly higher risk of developing primary graft dysfunction [13,14]. Patients with *de novo* col(V)-specific CD4^+^ T cell reactivity arising after lung transplant were 10 times more likely to develop severe bronchiolitis obliterans syndrome (BOS) than col(V) non-reactive patients [10]. Most recently, we have shown an association between col(V)-specific responses and coronary artery disease (CAD) [12]. 

 Immunogenetic studies have found evidence for specific autoimmune disease-associated MHC class II haplotypes [15-19]. Interestingly, certain immunopathogenic peptides appear to be shared across different species. In Goodpasture’s syndrome, studies have shown cross-reactivity of the collagen type IV α3 chain non-collagenous 1 peptide SQTTANPSCPEGT, between rat MHC class II RT.1B and human HLA-DRA1*0101, DRB1*1501 (DR15) [20]. Currently, it is unknown whether such cross-species pathogenic epitopes of col(V) exist, or if certain HLA haplotypes are associated with susceptibility to or protection from col(V) autoimmunity or associated fibro-obliterative disease. The question also remains as to whether only those peptides that bind to recipient HLA class II influence post-transplant autoimmune CD4 T cell responses, or whether donor HLA class II can also play a role in de novo autoimmunity. 

## Materials and Methods

### Ethical Considerations

Blood was obtained by venipuncture or by leukapheresis, following informed written consent in accordance with protocols approved by the human subjects Institutional Review Board at the University of Wisconsin-Madison. 

 All animals were housed and treated in accordance with the guidelines outlined by the National Institutes of Health, and the study protocol was specifically approved by University of Wisconsin-Madison School of Medicine and Public Health (SMPH) Animal Care and Use Committee. Measures to alleviate suffering in the case of an animal used for trans-vivo DTH footpad injection are not usually necessary, but animals are monitored closely after injections and a veterinary consult would be requested if any animal showed signs of pain or distress, per the approved ACUC protocol. 

### Human Subjects and Clinical Criteria

HLA typing was performed using SSP technology [21]; 1200 organ donors at the University of Wisconsin-Madison between 1999 and 2006 were used as a reference population for HLA-DR frequency analysis. Human PBMC for immunological monitoring were obtained from whole blood prepared using Ficoll-Hypaque density gradient separation. Samples were obtained from healthy controls (n=30, 14 of which were HLA typed), end-stage lung disease patients (n=99, which included 54 who were previously studied [13,14], plus 45 new subjects), and lung transplant recipients (n=54). For peptide specificity studies, PBMC from leukapheresis of col(V)-reactive CAD patients (n=2), lung disease (n=2) and lung and heart transplant recipients (n=1 each) were used as a source of responder cells for *in* *vivo* (trans-vivo delayed hypersensitivity (TV-DTH) mouse footpad) and *in vitro* analysis. Selection of patients for leukapheresis was based on col(V) reactivity ≥ 25x10^-4^ inches net footpad swelling in fresh PBMC samples. There was no selection based upon HLA-DR type. 

Severe BOS was diagnosed by a sustained drop in forced expiratory volume in one second (FEV_1_) to 65-50% (BOS 2) or below 50% (BOS 3) of maximum FEV_1_ established within the first year after transplantation [22]. 

### Trans-vivo DTH Assay

The TV-DTH assay was used to test the immunological activity to different collagens and collagen-derived peptides, as described previously [10,12,23,24] Briefly, 6-10 million human PBMC were injected with antigen into footpads of naïve CB17-SCID mice in 20-40 µL volumes. Footpads were measured pre-injection and 24 hours after injections by dial thickness gauge (Mitutoyo, Kawasaki, Japan). Post-injection measurements were subtracted from pre-injection measurements to obtain a specific footpad swelling in units of 10^-4^ inches. To control for individual PBMC differences, we subtract background response (swelling response from injection of PBMC alone, which is typically 10-20 X 10^-4^ inches), from PBMC co-injected with antigen response. 

After normalizing each response to background, a strong immune response to specific antigen were considered to be those ≥25 X 10^-4^ inches net swelling and very strong responses were those >25 x 10^-4^ inches. For pre-transplant analysis of the immunogenetics of the col(V) response, we used the former criterion (≥25) to define a col(V) responder. For post-transplant analysis, a patient who at any time between 0.5-7.5 years after receiving a lung allograft had tested positive for col(V) reactivity by the latter criterion (>25), was considered a col(V) responder. These criteria are the same as previously used for analysis of pre-transplant risk for primary lung transplant dysfunction [14], and for post-transplant risk of BOS [10].

Similar methods were used to confirm the col(V) peptide specificity of T cell responses in col(V) immunized HLA-DR transgenic mice. Splenocytes and inguinal lymph node (ILN) cells were harvested and pooled from transgenic mice two weeks after immunization (see below) and injected with or without antigen into the footpads of naïve recipient mice for TV-DTH. CB17-SCID mice were used as TV-DTH recipient mice. Net swelling values in response to col(I), col(V) or to selected α1(V) peptides were determined in groups of three or more mice. P values for comparisons between responses of different HLA-DR Tg mice were determined by Mann-Whitney U test. 

It should be noted that responses to α1(V) peptides, whether detected in PBMC of col(V)-reactive patients or in spleen/lymph node of col(V)-immunized HLA-DR transgenic mice, could not be subject to any standard or cutoff value, since we did not know what proportion of the col(V) response we could detect with a single peptide. 

### Intracellular Cytokine Staining (ICCS)

We have previously shown the production of IL-1β and TNFα cytokines by monocytes after 5 hours of stimulation with col(v) [10]. As an alternative readout of col(V) epitope specificity, induction of intracellular cytokine staining (ICCS) of monocytes was measured at 16 hours. 1x10^6^ PBMCs from patients were plated onto 96-well plates and incubated overnight with media, or 5 μg of col(I), col(V) or DR/DQ restricted peptides in the presence of Brefeldin A ([10nM] Ebioscience, Beverly, MA). After 16 hours, cells were stained with surface markers CD3 (BD Biosciences, 555916) and CD14 (Biolegend, 325608), followed by fixation and permeabilization (Becton Dickinson, Lyse/Fix Buffer). After fixation and permeabilization, cells were stained with antibodies to IL-1β (BD Biosciences, 340516) and TNFα (BD Biosciences, 557647) followed by fixation with 2% paraformaldehyde. All samples were acquired on a FACScaliber flow cytometer and populations of IL-1β and/or TNFα positive cells amongst CD3^-^ CD14^+^ subsets were determined with FlowJo analysis software (Treestar). 

### Dose of protein and peptides

Human or bovine col(V) (both gifts from Dr. David Brand, Memphis, TN) was injected into CB17-SCID footpads with PBMC or mouse cells, respectively. Synthetic peptides were injected with human PBMC (1.0 μg per injection) or with immunized mouse cells (20.0 µg per injection). Purified col(I) or col(V) was injected at a dose of 5.0 (human) or 20.0 (mouse) µg per injection.

### Mice and Immunizations

All mice used in immunization studies and as adoptive hosts for TV-DTH assays with human PBMC were bred at the University of Wisconsin-Madison. Previously described murine MHC class II deficient HLA transgenic mice expressing HLA-DRB1*1501.AE^o^ (DR15) [25] were provided by Dr. Chella David (Mayo Clinic, Rochester, MN). HLA-A2.01/HLA-DRB1*0101.AE^o^ (A2/DR1) transgenic mice were also previously described [26] and provided by François Lemonnier (Institut Pasteur, France). HLA-DR15, and HLA-A2/DR1 transgenic mice were immunized subcutaneously into each inguinal pouch region with 120 µL of emulsion with Complete Freund’s Adjuvant (CFA) and 100 µg bovine col(V) (gift from David Brand) at a 1:1 ratio. All animals were housed and treated in accordance with the guidelines outlined by the University of Wisconsin-Madison and National Institutes of Health.

### Peptide-MHC class *II* Binding Assay

Binding of α1(V) peptides to recombinant HLA-DRA*0101, DRB1*0101 (DR1), DRB1*1501 (DR15), DRB1*0301 (DR17), DQA1*0501, DQB1*0201 (DQ2), DQA1*0102, DQB1*0602 (DQ6), and H-2 I-A^b^ was performed by ProImmune (Oxford, UK) using their cell-free MHC Class II REVEAL binding assay. This method identifies neoepitopes based on conformational changes induced in MHC class II molecules upon binding a peptide. MHC class II/neoepitopes are recognized by a conformation- specific labeled antibody to provide a binding readout. A library of 101 15-mer peptides, with 5 amino acid (AA) overlaps, spanning the triple helical domain (1013 AA; p559-1572) of the α1(V) chain was generated and screened. Preliminary post-translational modification analysis indicated that certain prolines and lysines within the helical region of the α1(V) collagen were hydroxylated (HYP-hydroxyproline and HYL-hydroxylysine, respectively), and the peptide library that was prepared and screened for MHC class II binding incorporated the HYP, but none of the predicted HYL residues. The final post-translational modification analysis differed slightly [27]; however, the majority of *in vivo* analyses were performed with unmodified peptides. Peptide binding scores for each MHC class II protein were compared to 2 known positive control peptides for that allele. The highest binding positive control was assigned an arbitrary score of 100, allowing each individual test peptide to be assigned a score [0-650] relative to the highest positive control value.

### Synthesis of select α1(V) Peptides

Peptides identified as binders to recombinant HLA-DR1, -DR15, -DQ2, -DQ6, or mouse H-2 I-A^b^ in the *in vitro* binding assay were synthesized in Dr. Samuel Gellman’s Lab, University of Wisconsin-Madison. Briefly, peptides were synthesized on a Symphony automated synthesizer (Protein Technologies, Inc.) using Fmoc chemistry. Purification was done using HPLC and peptide analysis was performed using MALDI-TOF Mass Spectrometry to determine the correct peptide sequence. Peptides with HYP residues were synthesized with the most common 4-HYP; non–HYP versions of each peptide were also prepared. Peptides isolated at >95% purity were dissolved in DMSO (25 mg/ml stock) and diluted in PBS for use in subsequent experiments. 

### Statistics

Chi-square, Fisher’s exact tests and multivariate analysis were done with SAS/STAT (SAS Institutes). Mann-Whitney U test and ANOVA was performed with Prism 5.0 (GraphPad Prism software, La Jolla, CA).

## Results

### HLA-DR15 is associated with pre-transplant col(V) reactivity in patients with end-stage lung disease

**Figure 1:**
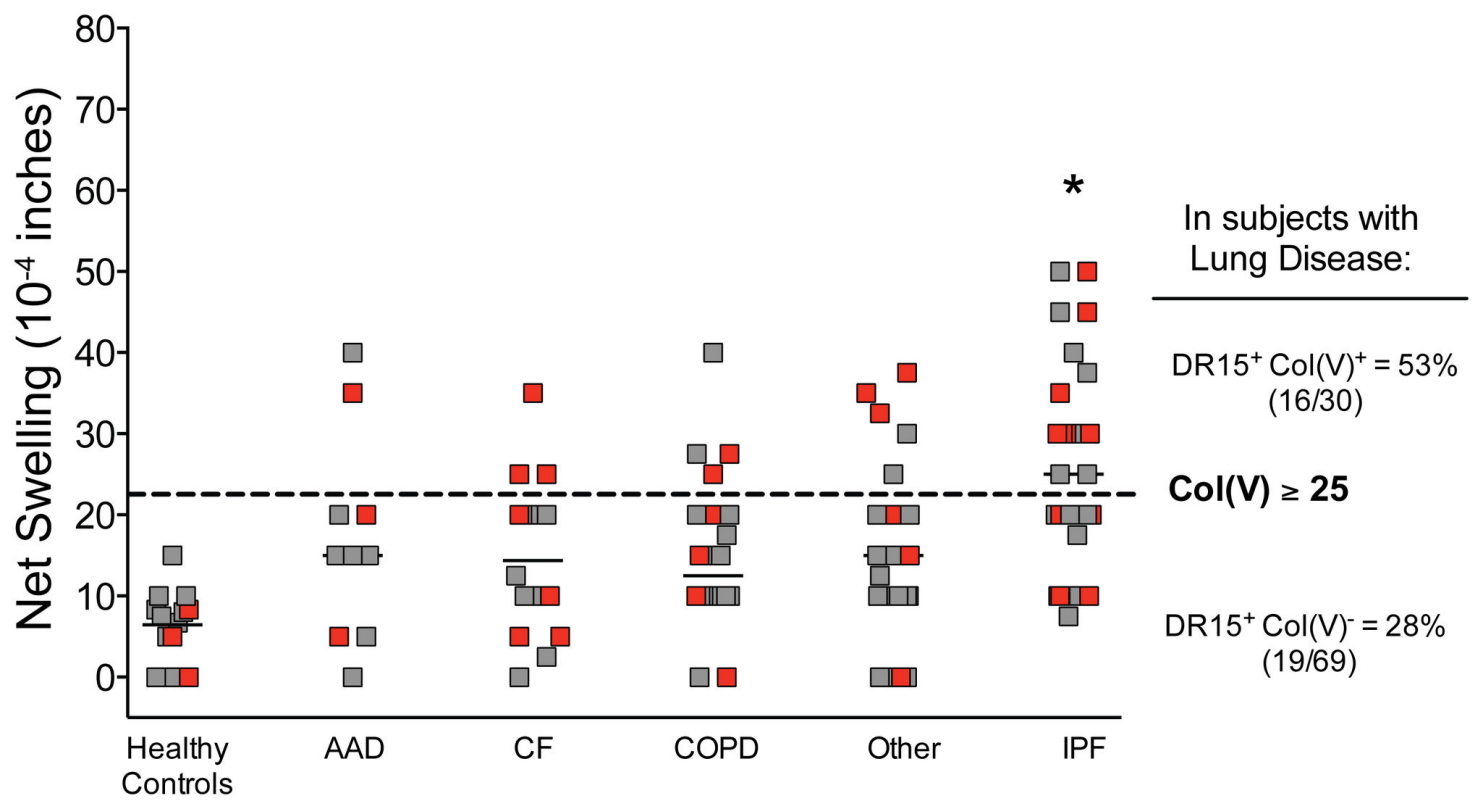
Differences in TV-DTH reactivity pre-transplant to col(V) in patients with end-stage lung disease by disease type and by prevalence of DRB1*1501. A significant number of patients with IPF had col(V) reactivity (net swelling ≥ 25 x 10^-4^ inches, dotted line; *ANOVA p<0.0007). Independently, patients with DRB1*1501 (red symbols) were enriched in col(V) responders (net swelling ≥ 25 x10^-4^ inches, Chi Square p= 0.01). AAD - α1 -antitrypsin disease; CF - cystic fibrosis; COPD - chronic obstructive pulmonary disease; IPF - idiopathic pulmonary fibrosis. Patients with lung diseases: n=53 (previous publication, [14]), n=45 new subjects, and n=1 subject who donated samples to both studies (average values of 2 time-points). Healthy subjects (n=14) with known HLA-DR types were tested and used as controls.

Col(V)-specific T cell-mediated immunity was assessed by TV-DTH assay in 99 patients awaiting lung transplantation at University of Wisconsin-Madison Hospital and Clinics. As depicted in Figure 1 and Table 1, patients with idiopathic pulmonary fibrosis (IPF) were significantly more likely to have positive col(V)-specific cell-mediated immune responses than patients with other end-stage lung diseases (univariate ANOVA, p<0.0007). These responses were antigen-specific, as PBMC from the same end-stage lung disease patients failed to respond to control collagen types I or II (data not shown; see also 14). Normal healthy subjects universally had weak or no response to col(V) (Figure 1 and data not shown). 

**Table 1:** Univariate (chi square) and Multivariate analysis of associations with Col(V) reactivity >20x10^-4^ inches in TV-DTH in end-stage lung disease and prevalence of HLA-DR15.

**Variable**		**Total *n* (%)**	**Col(V)^+^*n* (%)**	**Hazard Ratio (Confidence Interval)**	**P value**
		99	30		
Univariate analysis					
Age					0.13
Gender					
	Male	46 (46)	19 (63)	2.68 (1.1-6.5)	**0.03**
Disease					
	AAD	10 (10)	2 (7)	0.54 (0.1-2.7)	0.45
	CF	16 (16)	3 (10)	0.48 (0.1-1.8)	0.27
	COPD	22 (22)	4 (13)	0.43 (0.1-1.4)	0.16
	IPF	30 (30)	16 (53)	4.50 (1.8-11.3)	**0.001**
	Other	21 (21)	5 (17)	0.66 (0.2-2.0)	0.46
HLA-DR Type					
	DR1	29 (29)	7 (23)	0.6 (0.2-1.7)	0.39
	DR13	23 (23)	5 (17)	0.6 (0.2-1.7)	0.31
	DR15	35 (35)	16 (53)	3.0 (1.2-7.3)	**0.01**
	DR17	23 (23)	4 (13)	0.4 (0.1-1.3)	0.12
Multivariate analysis					
	IPF			3.7 (1.4-10.2)	**0.009**
	HLA-DR15			2.7 (1.0-7.3)	**0.04**
	Gender (male)			1.8 (0.7-4.8)	0.2
	Age			1.0 (1.0-1.1)	0.4

 Univariate analysis of common HLA class II types in this patient cohort with lung disease also revealed that subjects with HLA-DR15 (Figure 1, red symbols) were more highly represented in the col(V)-responsive group (53% DR15^+^) versus non-responders (28% DR15^+^; Chi Square p=0.01, Table 1). As shown in Figure 1, healthy controls with HLA-DR15 (red symbols), did not have a higher response to col(V) than other HLA-typed controls. To exclude the possibility that the increased frequency of HLA-DR15 in col(V) responders was simply an indirect consequence of the recently reported association of HLA-DR15 with IPF [28], we conducted a multivariate analysis of risk for col(V) responsiveness (Table 1). This analysis demonstrated that HLA-DR15 and IPF disease were independent risk factors for developing pre-transplant col(V) autoimmunity, while gender (which had appeared to be significant in univariate analysis), age and weight were not significant multivariate risk factors. 

### HLA frequencies in col(V)-responsive transplant patients and lung donors

**Figure 2:**
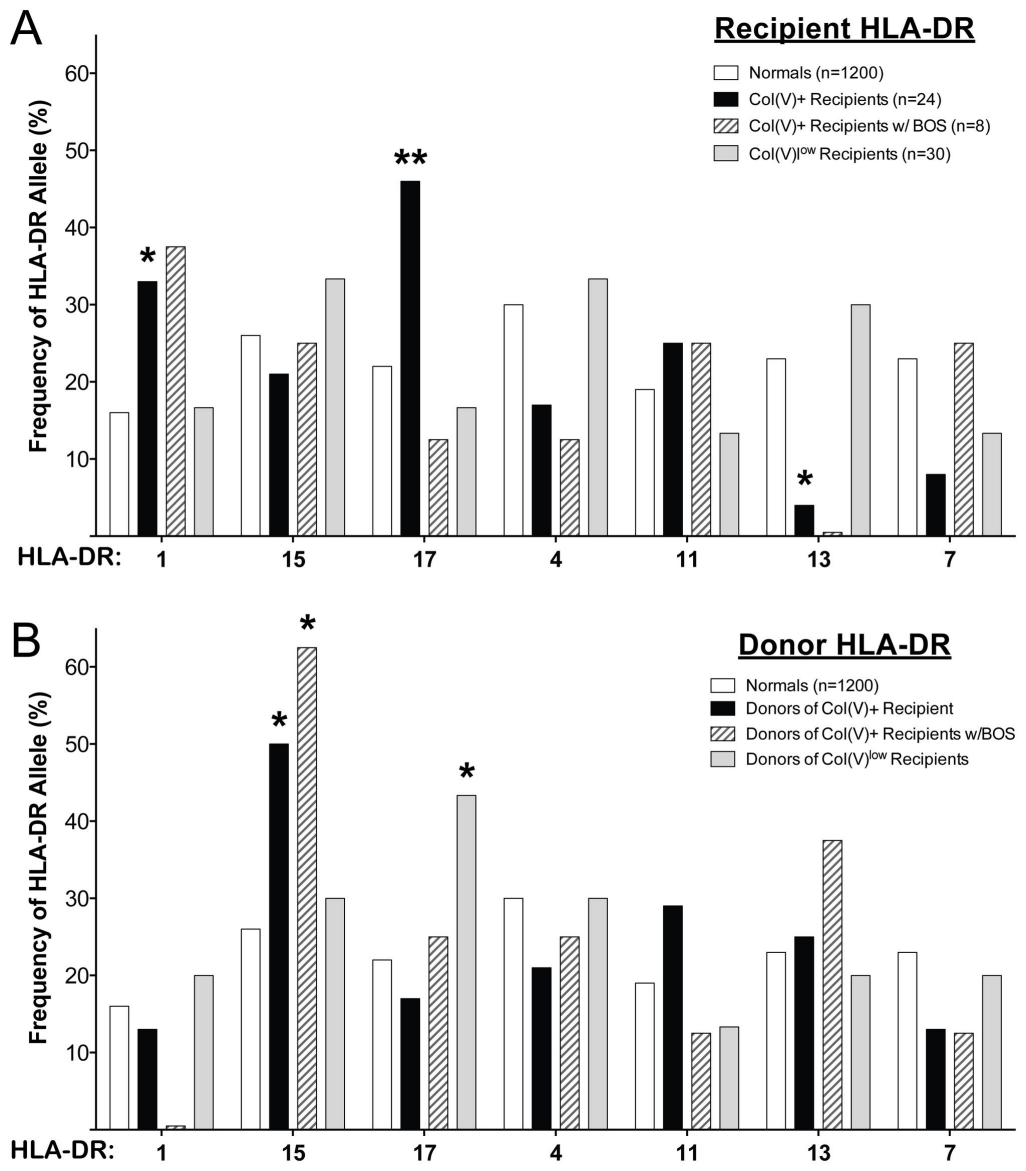
HLA-DR frequencies in col(V) reactive lung transplant recipients after transplantation. HLA-DR frequencies in very high col(V) reactive (TV-DTH net foot pad swelling > 25 x 10^-4^ inches) lung transplant recipients (A, black bars) or their donors (B, black bars) were compared to the HLA-DR frequency of controls (1200 subjects, open bars) and non-col(V) reactive or col(V)^low^ (≤ 25 x 10^-4^ inches) lung transplant recipients (A, grey bars) or their donors (B, grey bars). HLA-DR frequencies of very high col(V) reactive recipients (and their donors) who later developed BOS 2 or 3 (hatched bars) were also compared to HLA-DR frequency of controls. There were no significant differences in the frequencies of any HLA-DR alleles between normal control subjects from the University of Wisconsin (n=1200) and lung transplant recipients/donors (n= 281 recipients) or patients screened using the TV-DTH assay (n=54), data not shown. The frequency of HLA-DR1 (33%) and –DR17 (46%) was significantly higher in transplant recipients with very high col(V) responses (>25 x10^-4^ inches, A) than in normal controls (16% and 22%, respectively). HLA-DR13 was significantly less represented in col(V) responsive recipients compared to normal controls (4% vs 23% in controls). The frequency of HLA-DR15 was significantly higher in the donors of subjects who developed a high response to col(V) (50%) as well as in the donors of subjects with BOS 2/3 incidence (63%) compared to the frequency in the normal controls (26%). The frequency of HLA-DR17 was significantly higher in the donors of patients that did not have very high col(V) responses. *=p< 0.05, **= p<0.01.

Next we asked whether there were any HLA-DR associations with post-transplant col(V) responsiveness. Baseline analysis of the entire pool of lung transplant recipients (n=281) and donors (n=278) at our center revealed no significant differences in HLA-DR frequencies compared with the HLA/Molecular Diagnostics Lab database of 1200 control individuals or with the 54 subjects tested for col(V) response post-transplant [10]. When focusing on the very highly col(V)-responsive post-lung transplant population (TV-DTH net swelling >25 x10^-4^ inches, n=24/54 patients [10]), the frequency of patients expressing HLA-DRB1*01 (HLA-DR1) or HLA- DRB1*03 (HLA-DR17 ) was significantly higher in col(V) responders than controls (Figure 2A, black bars compared to open). The patients whose col(V) response remained negative or low (≤ 25 x10^-4^ inches) throughout their transplant course were similar in DR distribution to the reference control group (Figure 2A, grey bars compared to open bars).

Also of note, there was a significantly (p=0.03) decreased representation of HLA-DRB1*13 in these col(V)-responsive recipients compared to controls and overall lung recipients, suggesting a resistance to col(V) sensitization in this subgroup. While most of the 14 HLA-DR17^+^ lung recipients developed a response to col(V) post-transplant (11 had responses >25 x10^-4^ inches, while 2 others had a response =25 x10^-4^ inches), only 1 went on to develop severe (grade 2 or 3) BOS (Figure 2A, hatched bars). Importantly, there was no indication that HLA-DR15^+^ transplant recipients were more responsive to col(V) than other recipients, indicating that their high responses had been quelled in the post-transplant period

To test the possibility that the allograft itself might contribute to col(V) responsiveness and BOS risk, HLA-DR frequencies were also evaluated in lung donors (Figure 2B). HLA-DR frequencies in donated lungs transplanted into a recipient who became highly col(V)-responsive post-lung transplant were compared to the control HLA-typed individuals (Figure 2B, black bars compared to open bars). Remarkably, donors of transplants to highly col(V)-responsive recipients were significantly more likely to have a HLA-DR15 haplotype. These frequency analyses suggested that donor HLA-DR haplotypes and perhaps MHC class II^+^ donor APC, may contribute directly or indirectly to development of immune responses to col(V) post-transplant. In further support of a possible influence of donor HLA-DR type on transplant outcome, the majority (5/8 = 62.5%) of patients that went on to develop severe (grade 2 or 3) BOS and graft loss had received a transplant from an HLA-DR15^+^ donor (Figure 2B, hatched bars). In the patients whose col(V) response remained negative or low (≤ 25 x10^-4^ inches) throughout their transplant course (Figure 2B, grey bars), their donor DR distribution was similar to the reference control group (Figure 2B, open bars) except for the HLA-DR17 haplotype, which was significantly higher in these patients with low col(V) reactivity compared to controls distributions.

### α1(V) epitope discovery based on high responder HLA-DR/DQ types

We have previously shown that patient CD4 T cell responses to col(V) as measured by TV-DTH assay are mainly directed towards the α1(V) collagen chain [12]. To identify the immunogenic regions and epitopes of col(V) recognized by T cells, we utilized the ProImmune cell-free MHC Class II REVEAL binding assay (ProImmune, Oxford, UK), as described in the Methods to screen three HLA-DR (-DR1, -DR15, -DR17) and two HLA-DQ (DR17-associated -DQ2 and DR15 -associated -DQ6) antigens for binding to 101 α1(V) peptides. Because the C57BL/6 mouse is used as a model in lung transplant [29,30] and cardiac atherosclerosis [12] studies, and to look for inter-species shared epitopes, we also screened the same α1(V) peptide library for binding to H2-I-A^b^, the mouse DQ-like MHC class II equivalent in the C57BL/6 strain.

**Figure 3:**
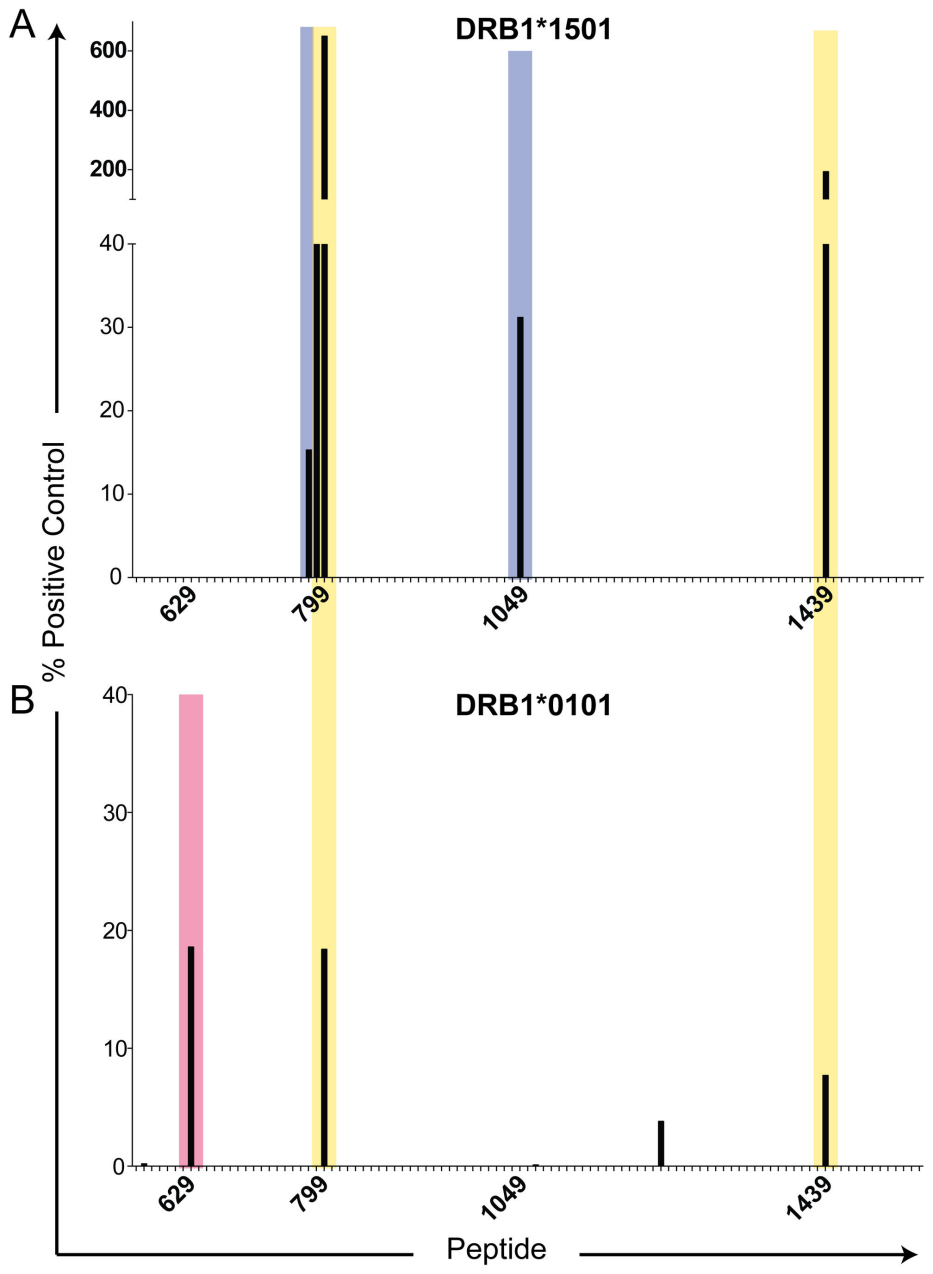
α1(V) peptide binding to HLA-DR1 and HLA-DR15. ProImmune REVEAL MHC-peptide binding assay screen of 5-amino acid overlapping peptide library (101 total peptides) of the α1(V) helical domain to HLA-DR15 (A), and HLA-DR1 (B). Each MHC:peptide binding score is based on known binding of a positive control peptide and a weaker binding positive control peptide. MHC:peptide binding scores above 12% are considered positive and have potential for immunological activity. Lack of bar for a peptide represents a score of 0%. Peptide number corresponds to the location in the α1(V) AA sequence. Blue shaded areas indicate epitope regions where only HLA-DR15 bound, red shaded areas where only HLA-DR1 bound, and yellow shaded areas indicate both HLA-DR1 and –DR15 bound within that helical domain.

**Figure 4:**
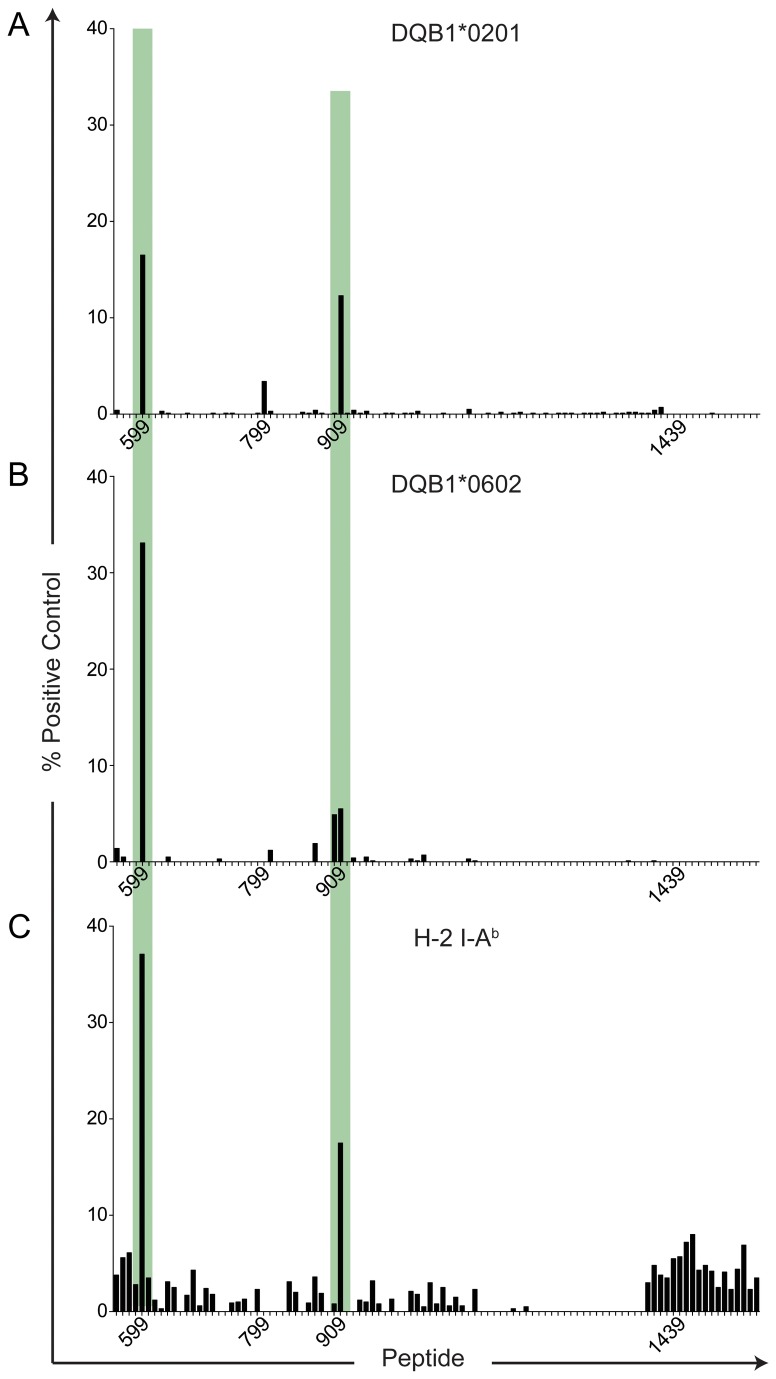
α1(V) peptide binding to HLA-DQ2, HLA-DQ6 and mouse H-2 I-A^b^. ProImmune REVEAL MHC-peptide binding assay screen of 5-amino acid overlapping peptide library (101 total peptides) of the α1(V) helical domain to HLA-DQ2 (A), HLA-DQ6 (B) and H-2 I-A^b^ (C). Each MHC:peptide binding score is based on known binding of a positive control peptide and a weaker binding positive control peptide. MHC:peptide binding scores above 12% are considered positive and have potential for immunological activity. Lack of bar for a peptide represents a score of 0%. Peptide number corresponds to the location in the α1(V) AA sequence. Green shaded areas indicate where HLA-DQ2, -DQ6 and H-2 I-A^b^ similarly bound within the helical domain.

 As shown in Figures 3 and 4, a total of 8 peptides were found to have potential immunological significance, with binding scores >12% of the positive control MHC-peptide binding. The sequences of these peptides are listed in Table 2, along with their binding scores. The MHC class II-binding peptides were present in six regions or “hot spots” located primarily in the N-terminal half (p559-1064) of the triple helical domain, with the exception of p1439, located near the C-terminus. Interestingly, no α1(V) peptides screened were found to bind to HLA-DR17, which was significantly over-represented in high col(V)-responders in lung transplant recipients post transplant, but under-represented in those patients who developed severe BOS (Figure 2A, black bar versus dark gray bar). Since HLA-DR17^+^ individuals tend to co-express HLA-DQA1*0501, -DQB1*0201 (HLA-DQ2) this suggests that the bulk of the reactivity to col(V) in these patients might be HLA-DQ2-biased. In Figure 3A, two peptides, p799 and p1439, bound to HLA-DR15 even more strongly than the positive control DR15-binding peptide (650% and 183% of control, respectively), while p789 bound almost as strongly as the positive control (94.2%). Four peptides exhibited single allele-specific binding: p1049 (KDGPPGLRGFPGDRG) only bound to DR15 (31.2% of control) as did overlapping peptides p779 (PPGPQGPIGYPGPRG) and p789 (PGPRGVKGADGIRGL (Figure 3A, blue) adjacent to the DR1/15 shared binding peptide p799 (Figure 3, yellow), while p629 (DRGFDGLAGLPGEKG) bound only to HLA-DR1 (18.2% control, Figure 3B, red). 

**Table 2:** Pan-Allele ProImmune REVEAL binding assay data for α1(V).

	**Position**	**AA Sequence [Human α1(V)]**	**Binding Score (% optimal)**		
**DR Binding Peptides**			DRB1*0101	DRB1*1501	DRB1*0301
	**p629**	DRGFDGLAGLP^‡^GEKG	18.6	0	0
	**p779**	PPGPQGPIGYPGPRG	0	15.3	0
	**p789**	PGPRGVKGADGIRGL	0	94.2	0
	**p799**	GIRGLKGTKGEKGED	18.4	**650**	0
	**p1049**	KDGPPGLRGFPGDRG	0	31.2	0
	**p1439**	LRGIPGPVGEQGLPG	7.7	**193.3**	0
**DQ-like Binding Peptides**			DQB1*0201	DQB1*0602	**H-2 IAb**
	**p599**	PP^‡^GPAGKP^‡^ GRRGRAG	16.5	33.1	37.1
	**p909** **	RGQRGPTGPRGERGP^‡^	12.3	5.5	17.5

‡ Position of 4-hydroxyproline in peptide made for binding assay

** Peptide synthesized in an extended form with an AG for the functional assays

It should also be noted that the identified α1(V) epitopes for HLA-DR1 and -DR15 were different from the epitopes shared between HLA-DQ2, -DQ6 and the mouse DQ homologue, H-2 I-A^b^ (Figure 4, green compared to Figure 3). All HLA-DQ/I-A^b^-peptide binding scores were below 38% of positive control (Table 2). Interestingly, none of the peptides identified by the *in vitro* binding assay were predicted based on the RANKPEP peptide binding prediction algorithm (http://imed.med.ucm.es/Tools/rankpep.html) (Table S1).

### Peptides identified by in vitro MHC-binding elicit T cell responses comparable to those elicited by intact col(V)

**Figure 5:**
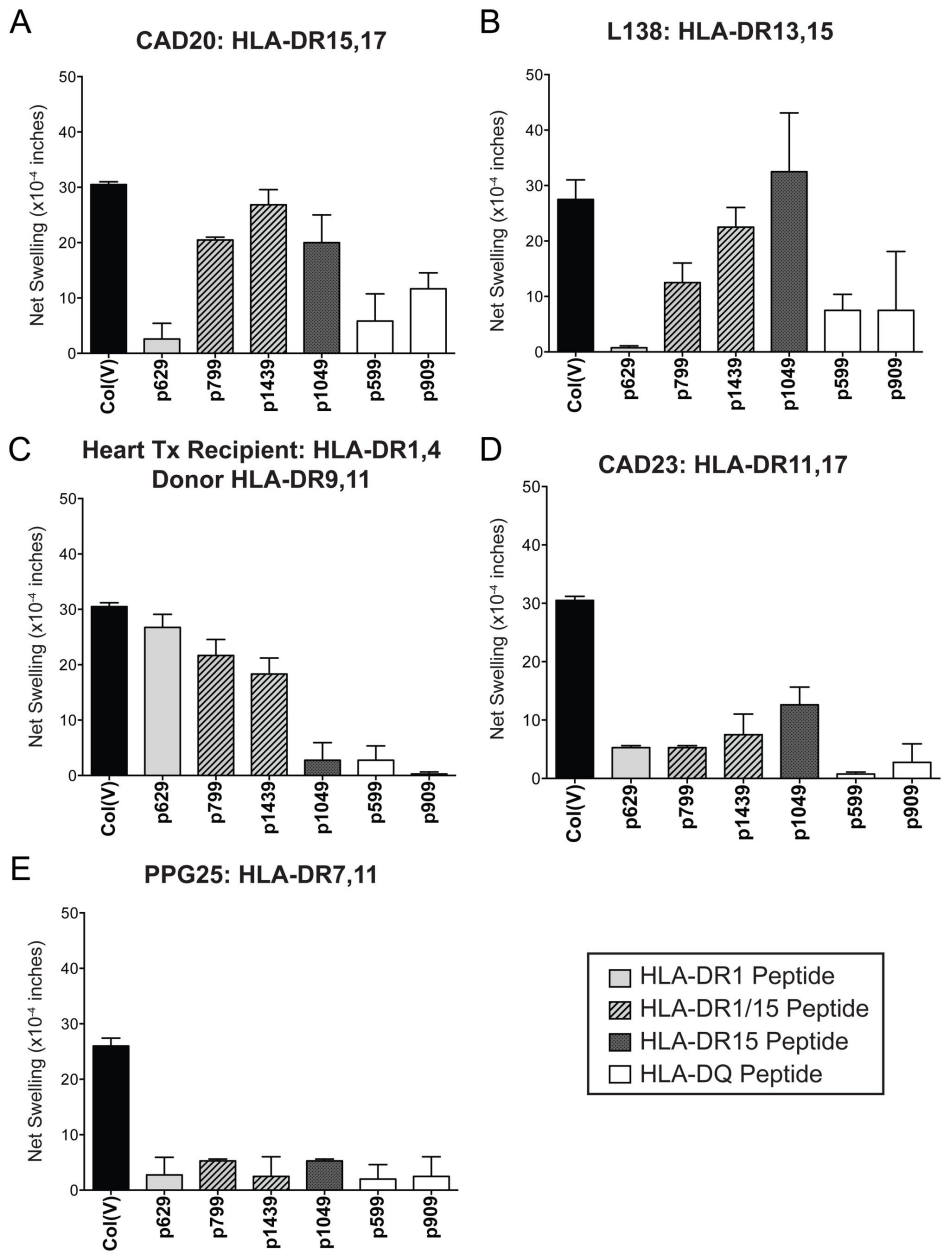
Candidate α1(V) peptide screen in col(V)-responsive patients. PBMC from (A) CAD patient 20 (HLA-DR15, 17; -DQ2,6), (B) End-stage lung disease patient L138 (HLA-DR13,15; -DQ5,6) (C) Heart transplant recipient H76 (HLA-DR1,4; -DQ3,5, with donor HLA-DR9,11; -DQ3,-), (D) CAD patient 23 (HLA-DR11,17; -DQ2,7) and (E) End-stage lung disease patient PPG25 (HLA-DR7,11; -DQ2,7) were screened for reactivity to ProImmune binding peptides by TV-DTH. Results from HYP and non-HYP p629, p599 were combined. Patient response to intact col(V) (black bar), HLA-DR1-specific peptide (light gray bar), HLA-DR1/15-specific peptides (hatched bars), HLA-DR15-specific peptide (dark gray bar), and HLA-DQ/H-2 I-A^b^ binding peptides (white bars) are shown. (Data represents the mean of 2-4 separate assays).

 HLA-DR and -DQ binding peptides were synthesized in both non-hydroxylated (all peptides), and hydroxylated proline (P* in peptides 599, 629, and 909 only; Table 2) forms and used for functional analysis, in order to look for possible correlations between peptide binding and T cell immunoreactivity. Figure 5 shows the α1(V) peptide reactivity of PBMC from col(V)-reactive patients compared to intact col(V) (black bar). PBMC from a col(V)-reactive HLA-DR15^+^ CAD patient, CAD20 (HLA-DR15,17, -DQ2,6), was screened for responses to α1(V) peptides by TV-DTH (Figure 5A). The HLA-DR15-specific peptide p1049 (dark gray bar) and -DR1/15 cross-reactive peptides p799 and p1439 (hatched bars) elicited strong swelling responses similar in magnitude to the response to intact col(V). A weak response was elicited by the HLA-DQ2/DQ6-specific peptide p909, while the DQ peptide 599, and the HLA-DR1-specific peptide, p629, generated low/no response (Figure 5A, light gray bar). Similar results were obtained with PBMC from another col(V)-reactive HLA-DR15^+^ patient, L138. This end-stage lung disease patient awaiting lung transplantation, had TV-DTH responses to the p1049 and p1439 that were as strong as the response to intact col(V), but did not respond to the HLA-DR1-specific peptide p629 (Figure 5B). PBMC of HLA-DR15^+^ healthy controls failed to respond to intact col(V) (Figure 1) or to HLA-DR15 binding peptides in TV-DTH assays (data not shown), confirming that active autoimmune response to col(V) is required for peptide reactivity.

Peptide screening in a DR1^+^ col(V)-responsive heart transplant recipient H76 (HLA-DR1,4; -DQ3,5; Figure 5C) confirmed the biological activity of the HLA-DRB1*0101-specific peptide, p629. This patient’s PBMC responded strongly to p629 and somewhat less to HLA-DR1/15 peptides p799 and p1439. Neither the transplant donor nor the recipient expressed HLA-DR15, and the patient’s PBMC were unreactive to HLA-DR15-specific peptide, p1049, as well as HLA-DQ-specific peptides p599 and p909.

The α1(V) candidate peptides were also screened with PBMC from two DR11^+^, col(V)-responsive patients lacking HLA-DR1 or -DR15. PBMC from patient CAD23 failed to respond to HLA-DR1-specific peptide p629, or to DR1/15 shared epitopes (Figure 5D). There was a weak response to the DR15- specific peptide p1049 (12.5 ± 2.5 x 10^-4^ in). Responses to the HLA-DQ2/DQ6- binding peptides were also low in this patient. PBMC from an HLA-DR-7, 11 expressing col(V)-reactive patient awaiting transplantation, PPG25, were also screened for responses to the same series of peptides but none were detected (Figure 5E). 

**Figure 6:**
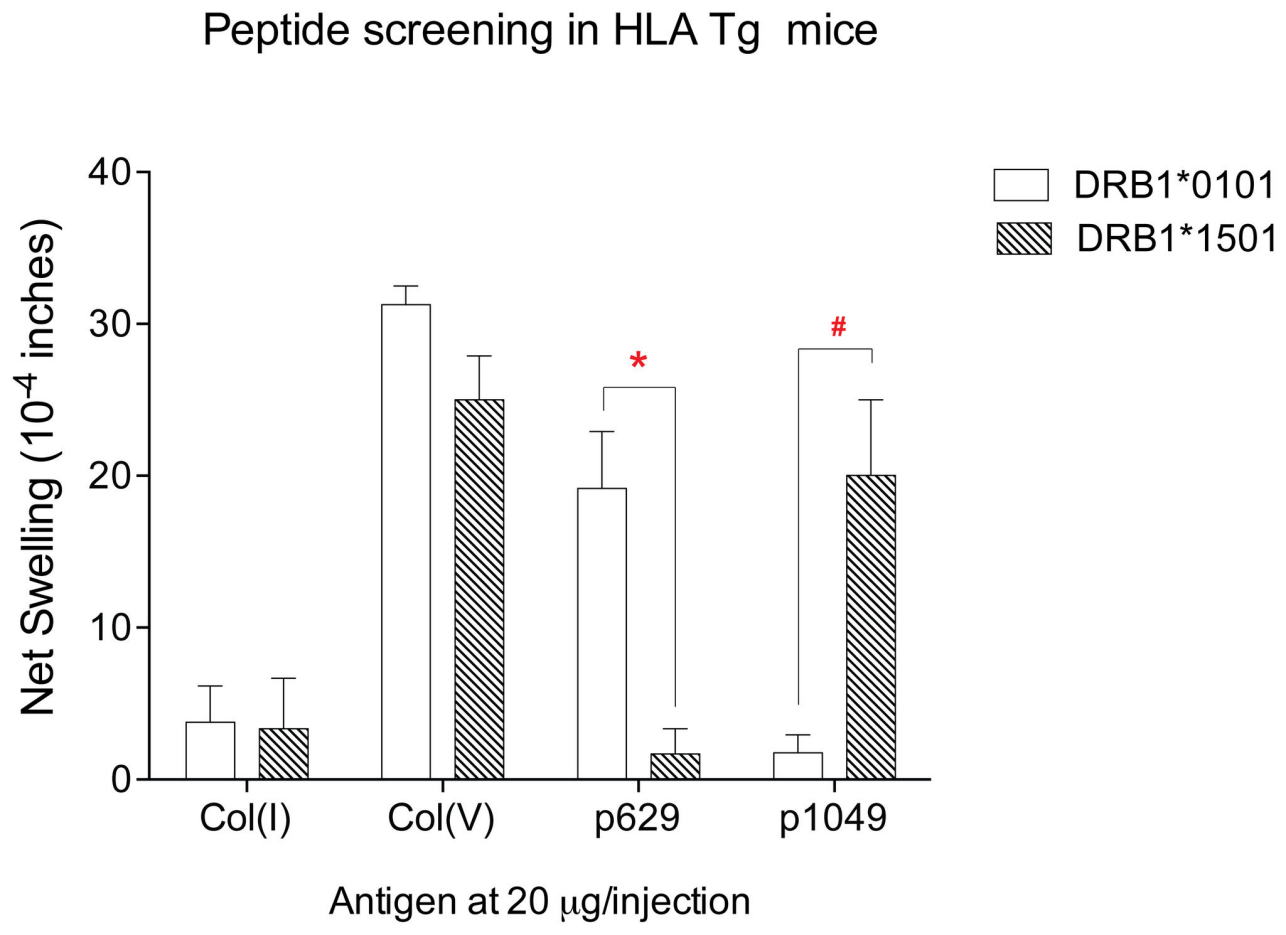
Candidate α1(V) peptide screen in col(V) immunized HLA-transgenic mice. Splenocytes and ILN cells from col(V) immunized HLA-A*0201/DRB1*0101 Tg (open bars) and HLA-DRB1*1501 Tg (hatched bars) mice were pooled and co-injected with col(V), col(I), or candidate α1(V) peptides into footpads of CB-17 SCID mice to determine peptide-specific swelling responses ; n=3-6 mice were used for each test. * p=.026; # p=.057 comparing peptide responses in DRB1*0101 vs. DRB1*1501 Tg mice using an unpaired Mann-Whitney U test. Responses to col(I) negative control and col(V) positive control were not significantly different between the two Tg mouse strains.

To confirm biologic activity and specificity of the HLA-DR1 and -DR15 binding α1(V) peptides p629 and p1049, we tested mice deficient for expression of mouse MHC class II and transgenic for either HLA-DRB1*0101 or HLA-DRB1*1501 (Figure 6). Mice were immunized with bovine col(V), and 2 weeks later spleen and inguinal lymph nodes were pooled and assayed by TV-DTH. Col(V)-immunized HLA-DR1 (Figure 6, open bars) and HLA-DR15 (Figure 6, hatched bars) Tg mice responded equally well to intact col(V); however, they differed markedly in their responses to the peptides. Peptide p629 elicited a response in col(V)-immunized DR1- but not DR15- Tg mice, whereas p1049 elicited a response in col(V)-immunized DR15- but not DR1- Tg mice. Col(V)-immunized C57BL/6 mice used as a control, responded to col(V) and the DQ/I-A^b^-specific peptide p599 but not to p629 or p1049 (data not shown). 

Taken together, the human and HLA-transgenic mouse T cell responses to α1(V) peptides confirm the epitope specificity as determined by peptide-specific binding to HLA class II antigens. However, the strength of T cell-mediated immune response was not proportional to the strength of the binding to a particular MHC class II allele (compare Table 2 with Figures 5 and 6).

### HLA-DR15 expressed by a lung transplant donor is implicated in the immune response to col(V) by a HLA-DR1^+^DR15^neg^ transplant recipient

**Figure 7:**
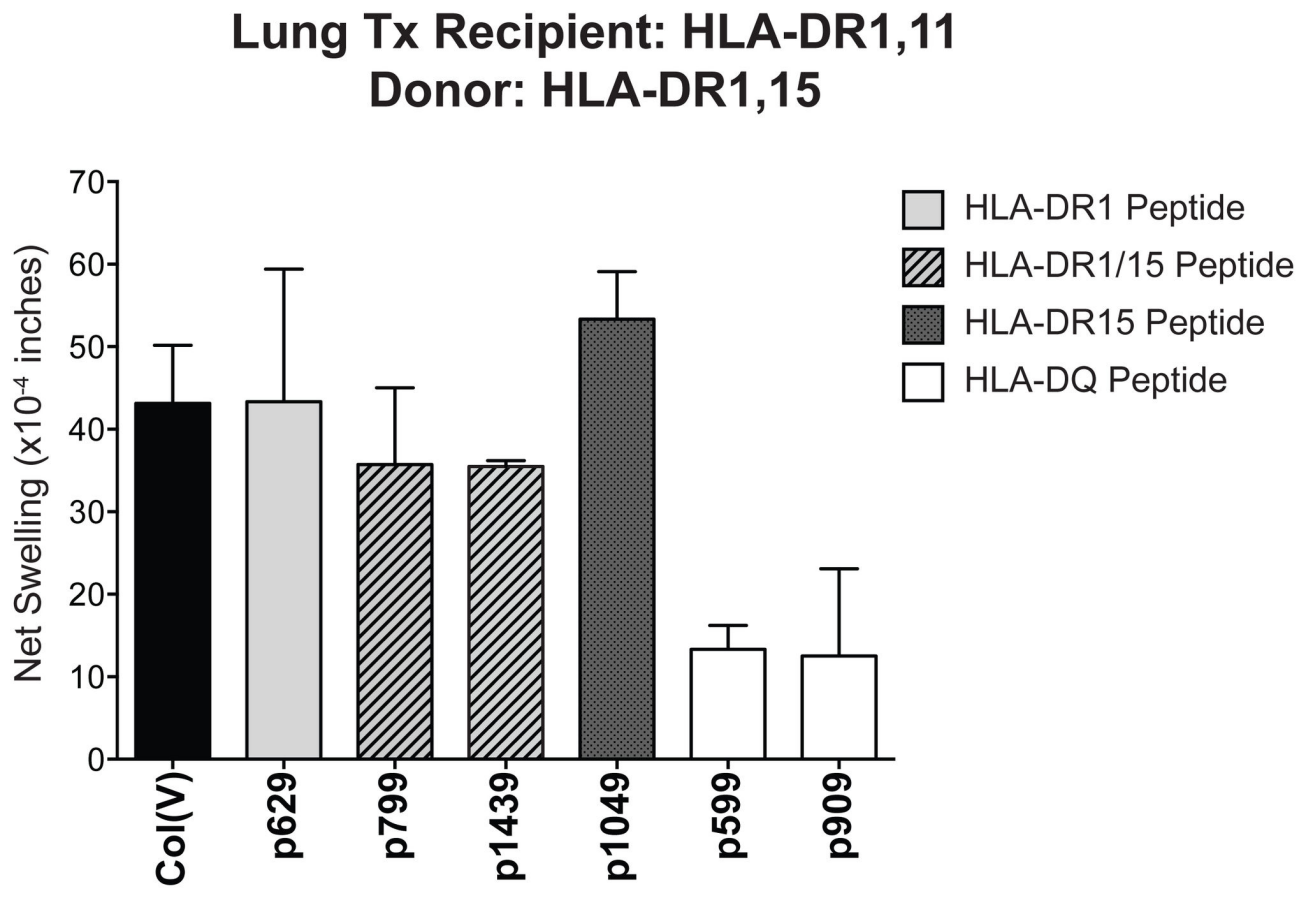
Unusual pattern of µ1(V) peptide response in a DR1^+^ lung recipient with a DR15^+^ transplant donor. PBMC from lung transplant recipient (L86, HLA-DR1,11; -DQ3,5, with donor: HLA-DR1,15; -DQ5,-) was assayed by TV-DTH for reactivity to α1(V) peptides or intact col(V). Results from HYP and prolinated p629, p599 were combined. Patient response to intact col(V) (black bar), HLA-DR1-specific peptide (light gray bar), HLA-DR1/15-specific peptides (hatched bars), HLA-DR15-specific peptide (dark gray bar), and HLA-DQ/H-2 I-A^b^ binding peptides (white bars) are shown. Data represents the mean of 2-5 independent experiments.

 In order to investigate the unusual association between HLA-DR15 expression by the lung transplant donor, and the presence of a strong immune response to col(V) in the lung transplant recipient (Figure 2B), we analyzed a lung transplant recipient patient who was negative for HLA-DR15 (recipient: HLA-DR1,11, -DQ3,5) but was transplanted with an HLA-DR15^+^ donor lung (donor: HLA-DR1,15, -DQ5,6). As shown in Figure 7, at seven years post-transplant patient L86 had a very high col(V)-specific TV-DTH response (43 ± 7 x 10^-4^ inches). A control α1(V) 15mer peptide, p150, located outside the triple helical domain, elicited no response (data not shown) while HLA-DQ-binding peptides p599 and p909 produced weakly positive swelling responses (Figure 7). The HLA-DR1 binding peptide, p629, as well as the HLA-DR1/15 cross-reactive peptides p799 and p1439, induced high TV-DTH responses, between 70-100% of the response to intact col(V) (Figure 7A, gray and hatched bars). Surprisingly, the HLA-DR15-specific peptide, p1049, induced the same degree of swelling as intact col(V) (Figure 7A, black bar). To confirm the unusual peptide reactivity pattern of lung patient L86, we used the induction of IL-1β in monocytes at 24 hours as a readout of antigen-specific T cell activity in whole PBMC cultures. As summarized in Table 3 (see also Figure S1), the responses of H76 and CAD20 tend to confirm the DTH data from Figures 5 and 6, showing that p629 and p1049 are indeed HLA-DR1, and –DR15 restricted, respectively. Importantly, the ICCS data obtained with PBMC of patient L86 confirmed the unusual “donor AND recipient” (p629 and p1049) HLA-DR-restriction of col(V) immunity in the lung transplant recipient. 

**Table 3:** Col(V) epitope analysis by induction of IL-1β^+^ in monocytes from PMBC after 24 hour *in* *vitro* culture.

Responder Source:		**CAD 20**	**Heart Tx H76**	**Lung Tx L86**
HLA-DR	Patient	**15**, 17	1,4	1,11
	Donor	n.a.	9,11	1,15
		**% IL-1β ^+^ of CD14^+^ cells in PBMC** ^a^		
Antigen	None	0.55	0.78	0.45
	Col(I)	0.8	1.03	0.49
	Col(V)	**2.71** ^b^	**3.2**	**2.61**
	p1439	**2.22**	**2.17**	**1.7**
	p1049	**1.7**	0.99	**2.12**
	p629	0.48	**1.92**	**1.85**
	p37 TE^c^	0.82	1.08	0.23

^a^ CD3^-^CD14^+^ cells were evaluated for production of IL-1β by ICCS.

^b^ Percentages in bold represent responses of ≥ 2-fold over media (no antigen) background

^c^ DSDAASPRTEPRAPWIEQ (aa37-54 of HLA-B7 heavy chain)

## Discussion

Epitope spreading, the phenomenon whereby initial autoimmune response to a particular immunodominant self-peptide is replaced by a second wave of response directed to a different epitope has been described extensively in mouse models of EAE [31]. The findings presented in this paper, particularly the data in Figures 2B and 7, and Table 3, suggest yet another way that epitope spreading may occur in lung transplantation, i.e. shifting from recipient HLA-DR -restricted epitopes, to donor HLA-DR -restricted epitopes. Based on HLA-DR1 and -DR15 epitope specificity analysis (Figures 3, 5, and 6), and the absence of strong peptide-specific responses in DR1 ^neg^15^neg^DR11^+^ col(V)-sensitized individuals (Figure 5D and E), it would seem unlikely that the HLA-DR1^+^,11^+^ patient L86 (Figure 7) would have been capable of responding to p1049, the DR15-specific epitope of α1(V), based on his inherited MHC class II antigens. Nonetheless, how there would be sufficient donor-derived HLA-DR15 in the patient’s PBMC, seven years after lung transplant, to allow for binding of p1049 and its presentation to T cells driving *in* *vitro* monocyte IL-1β production (Table 3) and *in vivo* tissue inflammatory responses (Figure 7) is unclear. Semi-direct pathway, the acquisition by recipient myeloid cells of donor MHC class II from the graft or via microchimerism, is one possibility [32]. We have recently found that the response to col(V)-derived peptides requires Th17 cells that interact with a monocyte antigen-presenting cell that presents the DR-bound peptide; the monocyte is thereby induced to express IL-1β in a P2X7 receptor-dependent manner [33]. Since both DR1-restricted and DR15-restricted antigen presentation to T cells apparently triggered IL-1β expression in the monocytes of a DR1^+^ recipient of a DR15^+^ lung transplant, it would imply that either there is donor chimerism or DR15 antigen acquisition within the monocyte population of patient L86. In any case, additional examples of donor HLA-DR driven changes in the epitope specificity of the col(V) response in lung transplant patients will be required to definitively test this novel hypothesis. 

During progression of chronic rejection, “inter-molecular” epitope spreading [i.e. presentation of peptide moving from one self MHC class II allele to another] in heart and kidney transplant recipients has been described for indirect pathway responses to donor alloantigens [34,35]. Perhaps the acquisition of a donor class II-restricted response to allo- or self-antigen is yet another extension of this normal progression.

It has recently been reported that HLA-DRB1*1501 is overrepresented in patients with IPF compared to normal subjects [28]. So far, we have not observed a HLA-DRB1*15 association with IPF in patients at the University of Wisconsin-Madison, but high resolution MHC class II typing has not yet been applied to our patient sample. Thus an association of IPF disease with HLA-DRB1*1501 remains a possibility. However, we did find that the two factors, IPF disease and HLA-DR15, were independently associated with increased pre-transplant anti-col(V) T cell responses in multivariate analysis (Table 1). IPF, like atherosclerosis [12], has a significant col(V)-specific autoimmune component [13,14]. Our data indicates that HLA-DR15 contributes to col(V) autoimmunity in IPF as well as in non-IPF end-stage lung disease. 

HLA-DR15 was also a significant risk factor for anti-col(V) responses after lung transplant, but surprisingly, only when HLA-DR15 was expressed in the healthy donor lung(s). This unusual finding suggests a role for donor cells and their MHC class II molecules in presenting self antigen-derived peptides to recipient T cells after transplantation. The number of patients who developed severe (grade 2/3) BOS in our study was small (n=8/54), but it is of interest that 5/8 had received lungs from HLA-DR15^+^ donor. In preliminary analysis of a group of 278 lung transplant patients at a single center, we have found a strong trend toward greater risk of severe BOS in those with HLA-DR15^+^ donors (p=0.08; Haynes, LD, Meyer, KC, Amir, A, Wilkes, DS, and Burlingham, WJ, manuscript in preparation). 

 In peptide binding studies, two α1(V) peptides, p799 and p1439, bound 6- and 2-fold, respectively, more strongly to HLA-DR15 than the positive control peptide. This was by far the strongest peptide binding observed in the ProImmune Reveal assay when compared to the five other MHC class II molecules tested. The unusually high affinity of col(V) peptides for the HLA-DR15 molecule could partly explain why HLA-DR15 was an independent risk factor associated with col(V) reactivity in patients with end-stage lung disease, and why having a DR15^+^ lung transplant might put a recipient at greater risk for developing anti-col(V) sensitization (Figure 2). 

 Interestingly, after lung transplantation, recipient HLA-DR1 and DR17, but not DR15, were associated with the development of *de novo* post-transplant col(V) reactivity. In our experience the high col(V) immunoreactivity observed pre-transplant appears to be markedly suppressed in post-transplant peripheral blood, which might explain why there was no DR15 bias to post-transplant col(V) responsiveness on the recipient side. The association of donor HLA-DR15 with delayed appearance of Th17 responses to col(V) and its associated BOS risk may also be related to a high binding affinity for col(V) peptides. For example, by adding new, higher affinity HLA class II molecules expressed by the donor organ, there is potential for epitope spreading, as some peptide/self MHC-reactive T cells in the host may be selected for cross-reactivity with donor class II-presented peptides that form highly stable peptide/MHC complexes. 

Cross-reactivity of normally self class II-restricted T cells with peptides presented by donor class II has been described for certain indirect pathway T cell clones that recognize allopeptides in the context of self MHC class II, but cross-react strongly with similar peptides presented in a donor MHC class II context [36,37]. The idea that “foreign” donor allogeneic MHC class II molecules can confer susceptibility or protection from autoimmune pathology in a host is also not entirely new—fetal-maternal microchimerism has been suggested as a source of foreign HLA class II influencing maternal post-partum susceptibility to rheumatoid arthritis [38,39].

 Interestingly, in lung transplant recipients who developed col(V) reactivity, we found that HLA-DR1^+^ patients trended toward a higher incidence of severe BOS [Figure 2A]. However, in the case of HLA-DR17, the trend was opposite, in that fewer DR17^+^ patients developed BOS, despite becoming col(V)-reactive. Our analyses also indicated patients that did not have col(V) reactivity post-transplant tended to have HLA-DR17^+^ donors at a higher frequency than the control population (Figure 2B, grey bar). This pattern of protection against BOS in HLA-DR17^+^ patients has been replicated in a preliminary analysis of our entire transplant database (p=0.05, Haynes et al., in preparation). These findings may relate to the fact that none of the α1(V) peptides screened could bind to the HLA-DR17 molecule (data not shown). HLA-DR17 transgenic mice lacking mouse class II did make T cell responses to intact col(V) (data not shown), but the inability of DR17 to form stable complexes with α1(V) peptides may limit pathogenicity of col(V)-specific DR17-restricted T cells. Alternatively, responses to α1(V) in HLA-DR17^+^ individuals might be due to peptides p909 and p599 presented by the closely linked HLA-DQ2. However, the latter explanation was not supported by the peptide responses of the HLA-DR17^+^ patient CAD23 (Figure 5D).

Using an “in silico” peptide prediction algorithm for HLA-DR4 and –DR7, Tiriveedhi et al. [40], proposed an "epitope spreading” phenomenon in T cell responses of lung transplant recipients during development of BOS, from α2(V) to α1(V) epitopes. However, no peptide/class II binding studies were performed to confirm the validity of the peptide prediction algorithm used. We have done such a comparison for the class II-binding α1(V) peptides identified in this study, and found no correlation with “in silico” analysis (see Table S1). Nonetheless, antibody data comparing pre-BOS vs. BOS time points supports the idea of progression from a “harmless” immune response directed to shared α1(V) and α2(V) epitopes, to a pathologic antibody response directed solely to private epitopes of α1(V) [40]. This agrees with studies of TV-DTH responses to col(V) showing that α1(V), not α2(V), is the sole target of the pathologic immune responses in atherosclerosis [12] and BOS [10]. The recently described induction of anomalous α1(V) gene expression and phenotypic changes in airway epithelial cells in response to IL-17 may be the basis of the immunodominance of α1(V) epitopes in BOS [29]. 

 Col(V) is one of the most highly post-translationally modified collagens [27]. The AA sequence used to generate the α1(V) peptide library was based on a preliminary analysis of post-translational modifications. Some prolines that were identified by mass spectroscopy as being modified by hydroxylation were incorporated as HYP residues in the generation of the α1(V) peptide library. However, no attempt was made to incorporate other modifications, such as the hydroxylated lysine (HYL) residues now known to occur at all 3 lysine positions in peptide p799, each of which are linked to the disaccharide glucosylgalactose [27]. Since p799 in its unmodified form yielded the highest-binding score to HLA-DR15 (650), and elicited nearly as strong a T cell responses as the intact triple helical domain of col(V), this extensive modification is clearly not necessary for antigen presentation to T cells. Our preliminary analysis of TV-DTH responses to HYP vs. proline-only containing versions of peptides p1439 and p629 did not show major differences in antigenicity (data not shown). However, previous studies of rheumatoid arthritis (RA) have indicated that other mechanisms of post-translational modifications, such as the enzymatic conversion of arginine residues to citrulline can indeed strongly impact the T cell autoimmune response [41-44]. Since all the α1(V) peptide epitopes listed in Table 2 contain one or more arginine residues, this same process could also affect immunogenicity of col(V) peptides. 

 In summary, the findings of a HLA-DR15 association with col(V) autoimmunity pre-transplant, and an association between donor HLA-DR15 and *de novo* anti-col(V) autoimmunity post-transplant, may have significance for the allocation of donor lungs and the treatment of lung transplant patients. The data imply that an HLA-DR15^+^ donor lung, though perfectly healthy at the time of transplantation, may over time impart increased risk for the development of col(V) autoimmunity and BOS. In this regard, the recent identification of gut microbiome differences between DRB1*0402 (RA-resistant) and DRB1*0402 (RA-susceptible) HLA-transgenic mice [45] raises questions as to the microbiome of the transplanted lung, and whether the DR15^+^ lung might contain microbiota that promote a Th17 response to the immunogenic HLA-DR15-binding α1(V) peptides. Discovery of the peptides described here, and others still to be found, may lead to therapeutic peptide delivery strategies to prevent BOS in recipients of a DR15^+^ lung transplant, as has been recently described for peptides that prevent EAE [46].

## Supporting Information

Figure S1 **Intracellular Cytokine staining for IL-1β supports response of lung recipient L86 to HLA-DR 15 specific binding peptides.** Cryopreserved PBMCs from patient L86 were plated at 1x10^6^ cells/well and treated with PBS, col(I), col(V), or the indicated DR- restricted peptide in triplicate for 16 hours in the presence of Brefeldin A. After stimulation, cells were stained for surface markers (CD3 and CD14), followed by permeabilization/fixation and subsequent intracellular staining for IL-1β and TNFα. Representative flow plots gated from CD3^-^CD14^+^ populations indicate that col(V), p1049, p1439 and p629 treatment leads to an increase in IL-1β positive cells, whereas the DQ restricted peptides, p37 TE (see Table 3) and p599 as well as col(I), fail to stimulate significant IL-1β production.
(TIF)

Table S1 **Highest ranked peptides based on RANKPep algorithm website.**
(DOCX)
