# Supplementary figures and images for: Epitope Analysis of the Collagen Type V-Specific T Cell Response in Lung Transplantation Reveals an HLA-DRB1*15 Bias in Both Recipient and Donor
Source: PLoS One. 2013 Nov 12;8(11):e79601. doi: 10.1371/journal.pone.0079601 (PMC3827168; doi:10.1371/journal.pone.0079601)

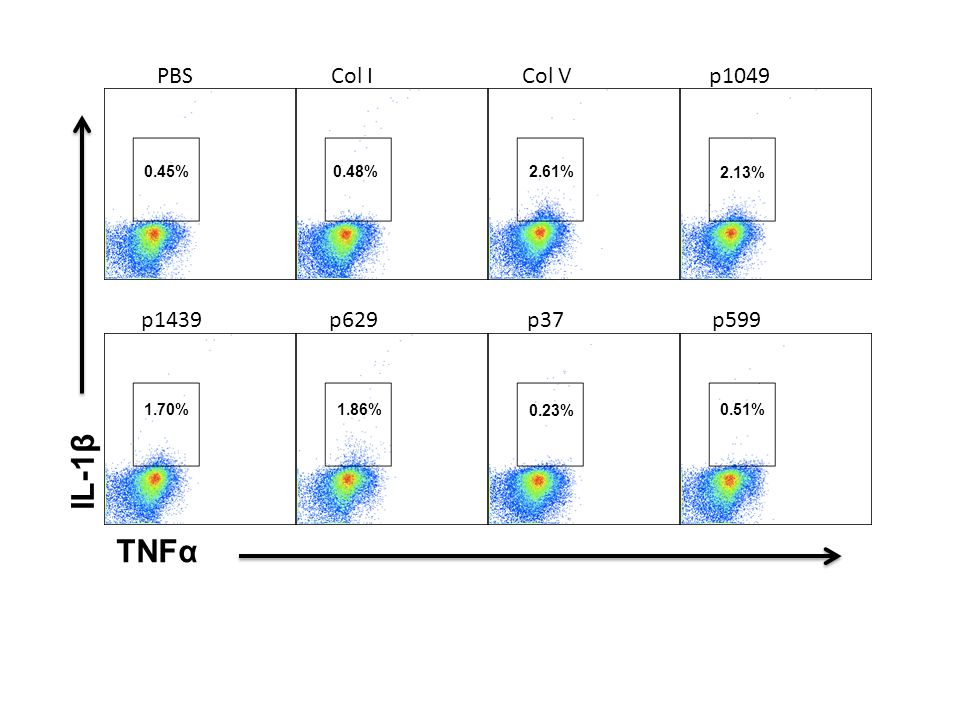

Supplement: Figure S1 — Intracellular Cytokine staining for IL-1β supports response of lung recipient L86 to HLA-DR 15 specific binding peptides. Cryopreserved PBMCs from patient L86 were plated at 1x10^6^ cells/well and treated with PBS, col(I), col(V), or the indicated DR- restricted peptide in triplicate for 16 hours in the presence of Brefeldin A. After stimulation, cells were stained for surface markers (CD3 and CD14), followed by permeabilization/fixation and subsequent intracellular staining for IL-1β and TNFα. Representative flow plots gated from CD3-CD14+ populations indicate that col(V), p1049, p1439 and p629 treatment leads to an increase in IL-1β positive cells, whereas the DQ restricted peptides, p37 TE (see Table 3) and p599 as well as col(I), fail to stimulate significant IL-1β production. (TIF) [file pone.0079601.s001.tif]
