# Supplementary material for: Epitope Analysis of the Collagen Type V-Specific T Cell Response in Lung Transplantation Reveals an HLA-DRB1*15 Bias in Both Recipient and Donor
Source: PLoS One. 2013 Nov 12;8(11):e79601. doi: 10.1371/journal.pone.0079601 (PMC3827168; doi:10.1371/journal.pone.0079601)
Supplement: Table S1 — Highest ranked peptides based on RANKPep algorithm website. (DOCX) [file pone.0079601.s002.docx]

| **Position** | **AA Sequence [Human α1(V)]** | **Analyzed for:** | **RANKpep Score (% optimal)** | **Binding Score (% optimal)** |
| --- | --- | --- | --- | --- |
| **p1063** | GLPGPVGALGLKGNE | DR1 | 18.3 | (0.0) |
| **p1075** | GNEGPPGPPGPAGSP | I-Ab | 18.8 | (2.5) |
| **p1198** | GEPGPRGQQGLFGQK | DR1 | 14.9 | (0.0) |
| **p1217** | PRGFPGPPGPVGLQG | I-Ab | **41.6** | (0.0) |
| **p1316** | PSGAAGPPGPKGPPG | I-Ab | **31.4** | (0.0) |
| **p1399** | GPEGRQGEKGAKGEA | DR1 | 5.0 | 0.0 |
|  |  | I-Ab | 0.0 | 4.8 |
| **p1476** | SGPKGEKGHPGLIGL | DR1 | 18.0 | (0.0) |
| **p1484** | HPGLIGLIGPPGEQG | DR1 | 0.0 | (4.8) |

Supplemental Table S1: Highest ranked peptides based on RANKPep algorithm website.

(Binding score) based on peptide closest in position to predicted peptide
